# The efficacy of a comprehensive lifestyle modification programme based on yoga in the management of bronchial asthma: a randomized controlled trial

**DOI:** 10.1186/1471-2466-9-37

**Published:** 2009-07-30

**Authors:** Ramaprabhu Vempati, Ramesh Lal Bijlani, Kishore Kumar Deepak

**Affiliations:** 1 Department of Physiology, All India Institute of Medical Sciences, New Delhi, India; 2 Sri Aurobindo Ashram, New Delhi, India

## Abstract

**Background:**

There is a substantial body of evidence on the efficacy of yoga in the management of bronchial asthma. Many studies have reported, as the effects of yoga on bronchial asthma, significant improvements in pulmonary functions, quality of life and reduction in airway hyper-reactivity, frequency of attacks and medication use. In addition, a few studies have attempted to understand the effects of yoga on exercise-induced bronchoconstriction (EIB) or exercise tolerance capacity. However, none of these studies has investigated any immunological mechanisms by which yoga improves these variables in bronchial asthma.

**Methods:**

The present randomized controlled trial (RCT) was conducted on 57 adult subjects with mild or moderate bronchial asthma who were allocated randomly to either the yoga (intervention) group (n = 29) or the wait-listed control group (n = 28). The control group received only conventional care and the yoga group received an intervention based on yoga, in addition to the conventional care. The intervention consisted of 2-wk supervised training in lifestyle modification and stress management based on yoga followed by closely monitored continuation of the practices at home for 6-wk. The outcome measures were assessed in both the groups at 0 wk (baseline), 2, 4 and 8 wk by using Generalized Linear Model (GLM) repeated measures followed by post-hoc analysis.

**Results:**

In the yoga group, there was a steady and progressive improvement in pulmonary function, the change being statistically significant in case of the first second of forced expiratory volume (FEV_1_) at 8 wk, and peak expiratory flow rate (PEFR) at 2, 4 and 8 wk as compared to the corresponding baseline values. There was a significant reduction in EIB in the yoga group. However, there was no corresponding reduction in the urinary prostaglandin D_2 _metabolite (11β prostaglandin F2α) levels in response to the exercise challenge. There was also no significant change in serum eosinophilic cationic protein levels during the 8-wk study period in either group. There was a significant improvement in Asthma Quality of Life (AQOL) scores in both groups over the 8-wk study period. But the improvement was achieved earlier and was more complete in the yoga group. The number-needed-to-treat worked out to be 1.82 for the total AQOL score. An improvement in total AQOL score was greater than the minimal important difference and the same outcome was achieved for the sub-domains of the AQOL. The frequency of rescue medication use showed a significant decrease over the study period in both the groups. However, the decrease was achieved relatively earlier and was more marked in the yoga group than in the control group.

**Conclusion:**

The present RCT has demonstrated that adding the mind-body approach of yoga to the predominantly physical approach of conventional care results in measurable improvement in subjective as well as objective outcomes in bronchial asthma. The trial supports the efficacy of yoga in the management of bronchial asthma. However, the preliminary efforts made towards working out the mechanism of action of the intervention have not thrown much light on how yoga works in bronchial asthma.

**Trial registration:**

Current Controlled Trials ISRCTN00815962

## Background

Although the efficacy of yoga in treating bronchial asthma[1] has been investigated since at least the 1960s [2-9], most of the previous studies have been uncontrolled, and have evaluated only a few selected yogic postures or breathing exercises. To the best of our knowledge, there is only one randomized controlled trial which has evaluated the efficacy of an integrated package consisting of yogic postures, breathing exercises, cleansing techniques, meditation, devotional sessions and lectures[10]. However, even this study did not investigate the mechanisms by which yoga improves the symptoms of bronchial asthma. The present randomized controlled trial was undertaken to study the efficacy of a comprehensive lifestyle modification and stress management program based on yoga in subjects having mild or moderate bronchial asthma. An attempt has also been made to monitor some immunological indicators of severity of disease and mast cell activation.

## Methods

### Subjects

The subjects were adult patients having mild or moderate bronchial asthma who were either referred to the Integral Health Clinic (IHC) of the All India Institute of Medical Sciences (AIIMS) by AIIMS physicians or came to IHC in response to our advertisements in local dailies.

The potential subjects went through a step-wise screening procedure. The inclusion criteria consisted of (1) age 18 years or older; (2) an established diagnosis of mild-to-moderate asthma for at least 6 months (meeting the American Thoracic Society[11] spirometry criteria for mild-to-moderate asthma, which requires either a forced expiratory volume in 1 second [FEV_1_]/forced vital capacity [FVC] below the lower limit of normal with a significant response to a bronchodilator [a ≥12% increase and a ≥200 mL absolute increase in FEV_1 _15 minutes after the administration of 2 puffs of a short acting *β*-agonist] or peak expiratory flow rate [PEFR] variability >20%); (3) taking at least one of the following: inhaled *β*-agonists, methylxanthines, anticholinergics, inhaled corticosteroids; and (4) stable medication dosing for the past month. Subjects were excluded if they (1) smoked currently (or in the past year) or had a smoking history of greater than 5 pack-years; (2) had a concomitant lung disease; (3) were taking leukotriene inhibitors or receptor antagonists, or mast cell-stabilizing agents for at least 6 months; (4) practiced yoga or any other similar discipline during 6 months preceding the study; (5) were pregnant; (6) had a chronic medical condition that required treatment with oral or systemic corticosteroids in the past month; (7) had a medical condition that contraindicated exercise; or (8) had an unstable medical condition.

Of the 138 potential subjects screened, 66 did not meet the above-described eligibility criteria. Of the 72 eligible subjects, only 60 were included in the study at the end of the 1-week run-in period and the other 12 subjects were self-withdrawn before randomization. The subjects completed the baseline measurements of all the outcome parameters after randomization. These 60 subjects were randomly allocated to either the yoga (intervention) group (n = 30) or the wait-listed control group (n = 30). However, one subject in the yoga group, and two subjects in the control group discontinued midway in the study. The results presented in this report are based on the data collected from only the 57 subjects who completed the study (yoga group, n = 29; control group, n = 28).

### Experimental Design

The present study is an open-label randomized controlled trial (RCT) with two parallel groups. The control group received conventional care whereas the experimental group received, in addition to conventional care, an intervention based on yoga. The subjects from both groups were on bronchodilators in the form of inhalers or inhaled corticosteroids depending on the judgment of the treating physician. None of the subjects was on systemic or oral corticosteroid therapy. However, subjects were not allowed to change their inhaled corticosteroid dose during the study period. During the study, the control group was offered a session on health education relevant to their illness, and at the end of the 8-wk study period, the intervention based on yoga. The outcome measures were assessed in both groups at 0 wk (baseline), 2, 4 and 8 wk. The subjects in neither group were encouraged to either delay or reduce their rescue medication use, but were instead advised to consult their treating physician for any dose changes, if required.

### Treatment Intervention

Participants assigned to the yoga group underwent a comprehensive yoga-based lifestyle modification and stress management program for 4 hours a day for 2 weeks. The sessions were conducted from 8 am to 12 noon. The subjects were neither unemployed nor disabled. Their ability to spend that much time in the clinic might be due to 'cultural differences'. In India, the patient respects his therapist and is willing to do whatever it takes to comply with the prescribed treatment. Corresponding to this attitude, the employer is also willing to bend the requirements of the job to make it possible for the employee to get the treatment. If the patient is a homemaker, other members of the extended family are willing to pitch in with childcare and other essential tasks to enable the patient to get the treatment. The program consisted of lectures and practical sessions on asanas (postures), pranayamas (breathing techniques), kriyas (cleansing techniques), meditation and shavasana (a relaxation technique). The 4-hour sessions included asanas and pranayamas for 1 hour; breakfast and building up of group support for 30 min; lecture and discussion for 2 hours and meditation for 30 min. The yoga practice sessions were conducted by a qualified yoga instructor. The lectures were on yoga, its place in daily life, its application to stress management, fundamentals of nutrition and health education relevant to their illness. Each subject received at least one session of about one hour of individualized counseling. During these individualized counselling sessions, the subject's questions regarding yogic practices, diet, prognosis, test results etc. were answered. In addition, family and social histories of the subjects were taken to establish a good rapport, and to discover any major mental stress, real or perceived, which might be aggravating the disease. In case of mental stress, some counselling, primarily in the form of cognitive restructuring based on the spiritual philosophy underlying yoga, was attempted. The counselling was done by physicians with special interest in yoga and mind-body medicine.

The physical practices consisted of elementary breathing exercises and loosening exercises followed by asanas under four categories (standing, sitting, prone and supine), pranayama, kriyas, and meditation based on raja yoga. Participants were instructed to follow a specific breathing pattern during each asana and asked to hold each pose in final position for up to 30 sec without holding their breath. Soothing instrumental music was played while the participants were practicing the yoga techniques to induce relaxation. Participants practiced yogic postures with awareness focused on their physical movements and breath. Each posture was followed by an appropriate relaxation technique for a short period. The classes concluded with deep relaxation in shavasana for about 15 min followed by guided imagery for about 5 min. The yoga practice sessions were about one and a half hour during the 2-wk training period and additional 6-wk home practice. The level of compliance for yoga practice was assured at least 5 days a week. The content of the daily home practice sessions was the same as during the 2-wk training, i.e. one hour of asanas and pranayama, 10 min of relaxation and 20 min of meditation. Subjects were provided printed material and audiocassettes to supplement live instruction. The yoga group subjects' compliance during the follow-up period was monitored by a diary on their adherence to yoga practice, dietary advice and rescue medication use, which they filled in everyday and brought during each visit. An individual yoga practice session was offered to the subjects during study evaluation visits. The control group was also asked to keep a diary of medication use and was attended by their treating physicians during study evaluation visits. During the follow-up period, telephonic support was also provided for motivating participants to improve their compliance.

The diet recommended for the yoga group was predominantly vegetarian; it mainly consisted of a combination of cereals and pulses, preferably unrefined; moderate amounts of judiciously chosen fat; moderate amounts of milk and milk products; moderate amounts of spices; vegetables and fruits about 500 g, vegetables being predominantly of the leafy green variety, and at least some eaten raw (uncooked).

### Outcome measures and techniques of measurement

The outcome measures were spirometric indices of pulmonary function, eosinophilic cationic protein as a marker of the course of the disease[12]; the degree of exercise-induced bronchoconstriction, urinary concentration of the prostaglandin D_2 _metabolite, 11β prostaglandin F2α before and after the exercise challenge as a marker of mast cell activation, quality of life and frequency of rescue medication.

#### Spirometric indices of pulmonary function

The indices of interest, viz. forced vital capacity (FVC), forced expiratory volume in the first second of expiration (FEV_1_), the ratio FEV_1_/FVC, peak expiratory flow rate (PEFR) and average forced expiratory flow rate during the expulsion of 25–75% of FVC (FEF_25–75%_) were measured by computerized spirometric equipment (K4b2, COSMED srl, Italy). The FVC manoeuvre was repeated at least thrice during each measurement. The highest of three acceptable readings of FVC and FEV_1 _were taken as the values for that sitting.

#### Eosinophilic cationic protein (ECP)

The serum concentration of ECP was assessed by sandwich ELISA kit (MBL ECP ELISA kit, Japan, code No. 7618 E). The coefficients of variation for inter-assay and intra-assay measurements were 10.7% and 11.1% respectively.

#### Exercise-induced bronchoconstriction (EIB)

The exercise challenge was given using a stationary bicycle ergometer (Bodyguard^® ^990, Sandnes, Norway). The workload was fixed at 80–85% of the maximum predicted heart rate while pedalling at 60 revolutions per min. The subjects were told to go on till they reach the limit of their dyspnoea or inability to continue the exercise for any reason. The duration of the exercise in different subjects ranged from 3–7 min. The heart rate was monitored continuously through a remote sensor attached to the subject's chest (Polar Electro Inc., Finland) and displayed on a computer screen. The exercise was performed in an air-conditioned room with ambient temperature between 24 and 27°C, and relative humidity between 40 and 50%. Thus the environmental conditions under which exercise was performed at different sittings were comparable. The degree of EIB was measured as highest degree of exercise-induced change in FEV_1 _from 0 min (baseline) to 3, 8, 15 and 30 min post exercise.

#### Urinary 11β prostaglandin F2α (11β-PGF2α)

Urine was collected 5 min before exercise and again 30 min after the end of exercise. Urine samples were stored, without the addition of any preservatives, at -20°C until analysis. The concentration of 11β-PGF2α excretion was estimated by using Enzyme Immuno Assay (EIA, Cayman Chemical, Ann Arbor, MI, USA; Catalog No. 516521). The inter-assay and intra-assay variations for 11β-PGF2α assay were 9.7% and 5.5% respectively. To correct for the effect of diuresis, creatinine concentrations were measured for all urine samples by a commercially available colorimetric assay (Wipro Biomed, Bangalore, India), using Jaffe' Rate method[13]. Therefore, the urinary 11β prostaglandin F2α concentrations were expressed as picogram per milligram of creatinine[14].

#### Quality of Life

Quality of life was measured by using a self-administered Asthma Quality of Life Questionnaire (AQOL) which is available in bilingual form, i.e. English and Hindi[15]. The AQOL is a 32-item disease specific questionnaire that has been validated to measure the problems that adult patients with asthma experience in their daily lives[16] and has also been validated on Indian population[17].

Subjects responded to each question on a 7-point scale (1 being maximum impairment; 7 being no impairment). The overall quality of life score is the mean score of all the 32 items. Thus the score may vary from 1–7. The 32 items are further grouped into four sub-domains viz., symptoms, activity limitation, emotional function and reactivity to environmental stimuli. The score for each sub-domain was also calculated as the mean score for items pertaining to that sub-domain. Thus, the score for each sub-domain may also vary from 1–7.

#### Rescue medication

The subjects were asked at the beginning of the study to add up how many times they had used a bronchodilator inhaler during the preceding 2-wk as prescribed by the physicians. For quantifying subsequent use, the subjects were told to make a note in their diaries every time they had to use rescue medication for getting quick relief from symptoms such as wheezing, cough or shortness of breath. At any point in time, the frequency of rescue medication was calculated as the average number of times that the medication had to be used in a day during the preceding 2-wk.

### Analysis of data

The baseline (0 wk) values of each outcome measure were compared with the values obtained at 2, 4 and 8 wk by Generalized Linear Model (GLM) repeated measures followed by post-hoc analysis[18]. Categorical variables were analyzed using Chi-square analysis. Continuous variables were analyzed using Mann-Whitney *U *test, Friedman test for overall group significance or the independent sample t-test, depending on the distribution of data. In case of outcome variables which changed in the same direction in both the interventional and control groups, between-group comparisons were also made to determine if the change in one group was significantly greater than that of the other group.

The degree of EIB was quantified by the percentage of highest fall in FEV_1 _reached after exercise during post-exercise measurements (3, 8, 15 and 30 min) as compared to the pre-exercise value. There was very high inter-individual variability in degree of EIB. Therefore the subjects were divided into exercise-sensitive (ES) and exercise-resistant (ER). Those who had a reduction of 15% or more in FEV_1 _post exercise at 0-wk were considered ES, and the rest ER.

The ECP values were log-transformed prior to analysis due to their wide range. Urinary 11β-PGF2α concentrations were not normally distributed and therefore expressed in median and inter-quartile ranges. The percentage change in urinary 11β-PGF2α following exercise was used for statistical analysis.

A change of ≥0.5 in the AQOL score has been considered the Minimal Important Difference (MID) as clinically meaningful difference[19]. Based on the MID, number needed to treat (NNT) was calculated using the method described by Gyuatt et al., (1998)[20] for trials with a parallel group design.

Differences were considered significant if *p *< 0.05. All statistical calculations were performed using the SPSS software 10.0.1 release for Windows (SPSS Inc, Chicago IL, USA).

### Ethical considerations

The protocol of the study was approved by the Ethics Committee of All India Institute of Medical Sciences. The subjects signed an informed consent form before being enrolled for the study and knew that they were free to withdraw from the study at any stage without assigning any reason.

## Results

The demographics and baseline values of outcome variables of subjects are given in Tables 1 and 2. As seen in these tables, the two groups are comparable at baseline in every respect except PEFR expressed as percentage of the predicted value.

**Table 1:** Demographics and Baseline Values of Outcome Variables

	**Yoga group** **(n = 29)**	**Control group** **(n = 28)**	***p*-value**
Age, years (mean ± SD)	33.5 ± 11.4	33.4 ± 11.5	0.96

Males, n (%)	13 (45)	20 (71)	0.29

Females, n (%)	16 (55)	8 (29)	0.19

Weight, kg (mean ± SD)	61.3 ± 11.5	58.9 ± 9.7	0.93

Height, cm (mean ± SD)	162 ± 10.1	161.8 ± 7.4	0.40

Body Mass Index (BMI) (mean ± SD)	23.4 ± 4.3	22.6 ± 4.0	0.44

Family history of asthma present, n (%)	13 (44.8)	11 (39.3)	0.67

Duration of Asthma, years (mean ± SD)	11.6 ± 9.5	10.5 ± 11.9	0.22

Mild asthma, n (%)	13 (44.8)	11 (39.3)	0.78

Moderate asthma, n (%)	16 (55)	17 (60.7)	0.82

FVC (% of predicted)	78.7 ± 13.3	75.2 ± 15.0	0.35

FEV1 (% of predicted)	70.2 ± 17.4	62.5 ± 19.2	0.11

FEV1/FVC (% of predicted)	80.4 ± 11.5	73.7 ± 14.8	0.06

PEFR (% of predicted)	68.6 ± 18.4	57.4 ± 19.7	**0.03**

FEF25-75 (% of predicted)	38.4 ± 14.5	33.9 ± 18.3	0.31

Serum ECP level (ng/mL)	1.32 ± 0.51	1.25 ± 0.43	0.89

EIB (%fall in FEV_1 _with EC)	15.0 ± 14.0	9.1 ± 10.8	0.16

Urinary 11β-PGF2α before exercise (pg/mg of creatinine), median (IQR)	455.4 (107.2–1098.1)	223.7 (98.8–562.7)	0.12

Δ Urinary 11β-PGF2α (pg/mg of creatinine) with exercise challenge, median (IQR)	26.9 (7.4–218)	38.4 (3.1–108)	0.55

AQOL total score	3.72 ± 1.17	3.64 ± 1.14	0.80

AQOL symptoms score	3.77 ± 1.34	3.62 ± 1.42	0.70

AQOL activity limitation score	3.66 ± 1.13	3.67 ± 1.17	0.95

AQOL emotional function score	3.94 ± 1.47	3.59 ± 1.39	0.35

AQOL environmental stimuli score	3.72 ± 1.17	4.04 ± 1.42	0.14

*Abbreviations*: AQOL, Asthma Quality of Life; FEV_1_, forced expiratory volume in 1 second; FEF_25–75_%, forced mid-expiratory flow between 25% and 75% of FVC; FVC, forced vital capacity; PEFR, peak expiratory flow rate; ECP, eosinophilic cationic protein; IQR, inter- quartile range; EC, exercise challenge; EIB, exercise-induced bronchoconstriction; 11β-PGF2α, 11β prostaglandin F2α.

*p*-value obtained by from Chi-square analysis or 't' test or Mann-Whitney *U *test depending on the type of variables.

All values are expressed as mean ± SD, except ECP expressed as log-transformed values and 11β-PGF2α as median with inter-quartile range.

**Table 2:** Daily dose of inhaled corticosteroids and rescue medication at baseline

*Type of medication*	**Yoga** (No. of subjects)	**Control** (No. of subjects)	***p*-value**
Mean inhaled steroid dose, μg/day (mean ± SD)§	320.4 ± 158.8 (n = 17)	360 ± 151.4 (n = 15)	0.48

Rescue medication use (puffs/day)¥	2.27 ± 1.5 (n = 26)	1.98 ± 2.09 (n = 20)	0.19

§ Daily dose of inhaled steroids in beclomethasone μg equivalents over the past 1-2 wk.

These were calculated on the assumption that beclomethasone 2000 μg = budesonide 1600 μg = fluticasone 1000 μg.

¥ Rescue medication = Inhaled rescue bronchodilator intake (puffs/day) use per day and an average for 2 weeks preceding the study visit.

p-value obtained from Chi-square analysis or Mann-Whitney *U *test.

**Figure 1:**
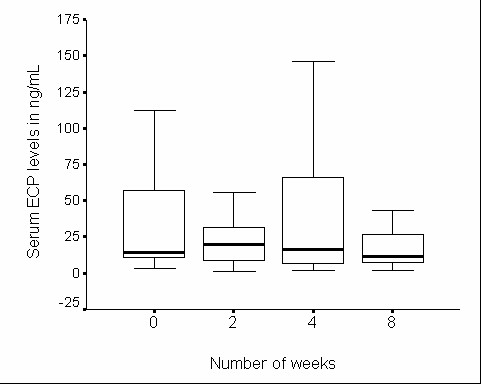
**Serum eosinophilic cationic protein in yoga group during the study**. Serum eosinophilic cationic protein in yoga group (n = 29) during 8 wk study period. Horizontal thick bars indicate median values and box the interquartile range (IQR).

**Figure 2:**
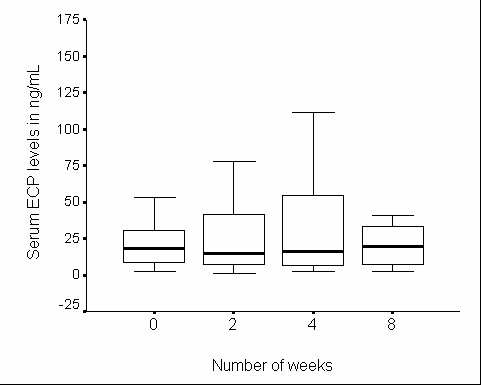
**Serum eosinophilic cationic protein in control group during the study**. Serum eosinophilic cationic protein in control group (n = 28) during 8 wk study period

**Figure 3:**
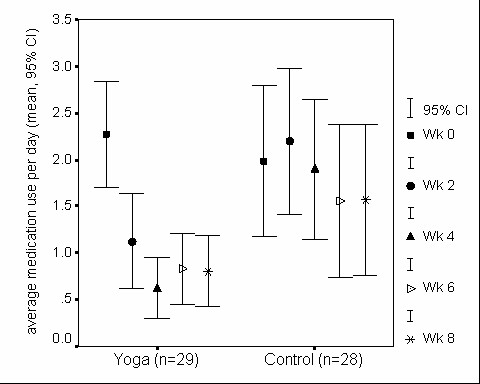
**Frequency of rescue medication use in yoga and control groups**. Rescue medication use was calculated as an average use of short acting β2-agonists per day (puffs/day) for 2 wk, hence, there are five time points. There is significant overall decline in rescue medication use in both yoga (p < .001) and control groups (p < .05). Also significant differences seen at 2 wk (p < .05) and 4 wk (p < .01) between yoga and control groups.

The values of the outcome measures at different time points during the study are given in Tables 3, 4, 5 and 6 and Figures 1, 2 and 3.

**Table 3:** Results of pulmonary function indices during the study

*Parameter*	*Group*	**Week 0**	**Week 2**	**Week 4**	**Week 8**	***p*-value**
**PEFR**	Yoga	68.6 ± 18.4	76.5 ± 20.5*	81.5 ± 20.9‡	85.3 ± 20.7‡	0.000
	
	Control	57.4 ± 19.7	58.2 ± 22.0	56.9 ± 26.2	56.2 ± 22.0	

**FEV1**	Yoga	70.2 ± 17.4	73.9 ± 19.6	76.1 ± 20.1	77.9 ± 17.2*	0.009
	
	Control	62.5 ± 19.2	63.1 ± 20.5	60.5 ± 21.6	59.9 ± 19.1	

**FVC**	Yoga	78.7 ± 13.4	78.0 ± 12.6	79.3 ± 13.0	82.2 ± 10.7	NS
	
	Control	75.0 ± 15.0	74.7 ± 18.3	73.4 ± 18.1	72.5 ± 17.5	

**FEV1/FVC**	Yoga	80.4 ± 11.5	82.6 ± 13.3	83.7 ± 13.4	83.1 ± 12.2	0.011
	
	Control	73.7 ± 14.9	74.8 ± 13.9	72.4 ± 15.1	73.3 ± 13.8	

**FEF_25–75%_**	Yoga	38.4 ± 14.6	42.0 ± 19.4	45.0 ± 20.5	45.0 ± 19.7	0.035
	
	Control	34.0 ± 18.3	33.8 ± 17.8	32.4 ± 18.1	31.1 ± 17.1	

All values are % of predicted and expressed as mean ± SD.

* p < .05; † p < .01; ‡ p < .001 based on post-hoc pair wise comparisons with 0 wk values.

*p*-values were obtained from the between groups comparison in the GLM, NS – not significant

**Table 4:** Exercise induced bronchoconstriction during the study

*Category*	*Group*	**Week 0**	**Week 2**	**Week 4**	**Week 8**	***p*-value**
% fall in FEV1 with exercise challenge	Yoga (n = 29)	15.05 ± 14.0	8.81 ± 12.9	5.65 ± 12.9	5.18 ± 11.8	0.005
	
	Control (n = 28)	9.13 ± 10.8	9.50 ± 11.1	10.28 ± 10.9	5.81 ± 10.1	NS

ES subjects (≥ 15% fall in FEV1)	Yoga (n = 13)	26.71 ± 12.7	13.33 ± 16.1	9.90 ± 14.5	7.95 ± 15.1	0.003
	
	Control (n = 9)	21.47 ± 6.4	8.83 ± 9.8	18.69* ± 8.2	12.71 ± 9.1	0.015

ER subjects (<15% fall in FEV1)	Yoga (n = 16)	5.58 ± 5.1	5.13 ± 8.4	2.20 ± 10.6	2.93 ± 8.0	NS
	
	Control (n = 19)	3.29 ± 6.6	9.82 ± 11.9	6.31 ± 9.8	2.53 ± 9.0	NS

All values are expressed as mean ± SD. ES – exercise sensitive; ER – exercise resistant.

*P*-value was based on Friedman test applied on % fall in FEV1 after exercise challenge within each group separately.

* Between groups comparisons were made by Mann-Whitney *U *test at individual visits, there was significant difference observed at 4 wk between groups in exercise-sensitive subjects (p = 0.025).

**Table 5:** Normalized values of Δ urinary 11β-PGF2α with exercise challenge

*Category*	*Group*	**Week 0**	**Week 2**	**Week 4**	**Week 8**	***p*-** **value^a^**
% change in 11β-PGF2α excretion. (All subjects)	Yoga (n = 29)	17.6 ± 18.6	19.7 ± 24.7	14.7 ± 16.5	16.8 ± 19.7	0.49
	
	Control (n = 28)	16.6 ± 21.0	18.6 ± 22.0	16.3 ± 22.3	20.7 ± 23.5	0.84

ES subjects (mean ± SD)	Yoga (n = 13)	18.3 ± 22.2	20.4 ± 29.5	13.8 ± 16.8	12.3 ± 16.9	0.63
	
	Control (n = 9)	15.5 ± 21.2	13.3 ± 18.1	28.5 ± 16.3*	18.9 ± 8.2	0.11

ER subjects (mean ± SD)	Yoga (n = 16)	17.1 ± 15.9	19.0 ± 21.0	15.4 ± 16.8	20.4 ± 21.5	0.79
	
	Control (n = 19)	17.0 ± 21.5	21.1 ± 23.6	10.6 ± 22.8	21.6 ± 28.2	0.08

All values (normalized) are expressed as % change in 11β prostaglandin F2α excretion with exercise challenge (mean ± SD). ES – exercise sensitive; ER – exercise resistant subjects.

^a^*P*-value was based on Friedman test in each group separately from % change in 11β-PGF2α with exercise.

*p < .05 based on Mann-Whitney *U *test at 4 wk between two groups.

**Table 6:** Asthma quality of life scores during the study

*AQOL sub domains*	*Group*	**Week 0**	**Week 2**	**Week 4**	**Week 8**	***p*-value**
Symptoms	Yoga	3.77 ± 1.3	5.07 ± 1.4‡	5.38 ± 1.1‡	5.42 ± 1.2‡	0.033
	
	Control	3.63 ± 1.4	3.96 ± 1.7	4.42 ± 1.5†	4.70 ± 1.7†	

Activity limitation	Yoga	3.66 ± 1.1	4.82 ± 1.3‡	5.21 ± 1.0‡	5.47 ± 1.1‡	0.033
	
	Control	3.67 ± 1.2	3.74 ± 1.5	3.90 ± 1.4	4.20 ± 1.5	

Emotional function	Yoga	3.94 ± 1.5	5.10 ± 1.6†	5.45 ± 1.3‡	5.71 ± 1.3‡	0.006
	
	Control	3.60 ± 1.4	4.01 ± 1.6	4.15 ± 1.8	4.32 ± 1.7	

Response to environmental stimuli	Yoga	3.46 ± 1.5	4.64 ± 1.5‡	5.0 ± 1.4‡	5.3 ± 1.6‡	NS
	
	Control	4.04 ± 1.4	3.83 ± 1.5	4.08 ± 1.6	4.40 ± 1.8	

Total Quality of Life	Yoga	3.72 ± 1.2	4.93 ± 1.3‡	5.28 ± 1.0‡	5.46 ± 1.1‡	0.013
	
	Control	3.64 ± 1.1	3.90 ± 1.5	4.17 ± 1.4*	4.50 ± 1.5†	

All values are expressed as mean ± SD.

* p < .05; † p < .01; ‡ p < .001 based on post-hoc pair wise comparisons with 0 wk values.

*p*-values were obtained from the between groups comparison in the GLM, NS – not significant.

There were no significant changes in pulmonary function indices in the control group during the course of the study. In contrast, there was a steady and progressive improvement in the subjects of yoga group during the same period. The change in the yoga group was statistically significant for FEV_1 _at 8 wk and PEFR at 2, 4 and 8 wk, as compared to the corresponding baseline values (Table 3). In addition, significant group mean differences were observed between yoga and control groups over the 8-wk study period in PEFR, FEV_1_, FEV_1_/FVC and FEF_25–75%_. In PEFR, since there is a significant baseline difference, baseline was considered as a constant covariate for between-group comparisons.

There was no significant change in serum ECP levels during the 8-wk study period in either group (Figures 1 and 2). The EIB decreased significantly in the yoga group, but not in the control group over the 8-wk study period (Table 4). Subgroup analysis in ES subjects revealed significant reduction in EIB over the 8-wk study period in both yoga and control groups but the amount of reduction observed was more marked in the yoga group than in the control group. In ER subjects the change in EIB was not significant in either group. Similar analysis of percentage change in urinary 11β-PGF2α levels in response to the exercise challenge revealed no significant change during the study in either group (Table 5). One interesting observation at 4-wk in the control group (in ES subjects) was that the EIB was rather significantly high, and this was associated with a significant increase in urinary 11β-PGF2α excretion in response to the exercise challenge as compared to the ES subjects in yoga group (Tables 4 and 5).

The AQOL scores showed an improvement over the 8-wk study period in both groups (Table 6). But the improvement was achieved relatively earlier and was more complete in the yoga group. At 2 wk, the improvement was statistically significant as compared to the baseline score in the total quality of life as well as each of the four sub-domains in the yoga group, but not in the control group. Even by 8-wk, significant improvement was seen in the control group only in total AQOL and symptoms but not in activity limitation, emotional function or the response to environmental stimuli. The NNT was found to be 1.82 for the total score, 2.41 for symptoms score, 1.66 for activity limitation score, 1.91 for emotional function score and 1.70 for the response to environmental stimuli score, which means ≤2 subjects needed to be treated with yoga plus conventional treatment for one subject to have a clinically meaningful improvement in quality of life over and above the improvement that the subject would have experienced with conventional treatment alone.

The frequency of rescue meditation use showed a significant decrease over the 8-wk study period in both the groups. But the decrease in rescue meditation use was achieved relatively earlier and was more marked in the yoga group than in the control group (Figure 3). Rescue medication use between yoga and control groups was significantly different at 2 and 4 wk because of the remarkable decrease in the frequency of the medication use only in the yoga group.

Although the level of compliance for yoga practice aimed at was at least 5 days a week, this level could not be sustained throughout the 8 weeks of the study. The compliance for yoga practice as reported by the subject was (average number of practice days per week): Wk 0, 4.6 ± 0.5; Wk 2, 4.6 ± 1.1; Wk 4, 4.5 ± 1.2; and Wk 8, 4.4 ± 1.1.

## Discussion

The present study has demonstrated that adding the mind body approach of yoga to the predominantly physical approach of conventional care results in measurable improvement in some subjective as well as objective outcomes in bronchial asthma. There have been previous RCTs on the efficacy of yoga which have arrived at similar conclusions[3,6,10]. But none of the previous trials have gone into the plausible mechanisms by which yoga might help bronchial asthma even in a small way as the present study has done. Since the major built-in difference between the two groups was the yogic intervention during 8-wk of the study, any difference in the outcome is likely to be due to yoga. The control being a wait-listed group, it does not control completely for non-specific effects of the experimental intervention such as expectation, belief, subject investment in time and effort, change in the subjects' daily routine, investigator interaction, social interaction and support, and placebo effect.

The 8-wk duration of the yogic intervention was an arbitrary compromise. It is long enough to demonstrate the effect, but the ideal would be a much longer study. However, in practice the decline in compliance increases steeply as the study gets longer, possibly owing to the time commitment required from the subjects[9]. Since there is no appropriate placebo possible for yoga, the subject cannot be blinded. The allocation of the subjects to the two groups was randomized, but the selection of subjects from the patient population was not random. Every subject who made it to our clinic to volunteer for the trial was already convinced in his mind about the beneficial effects of yoga because of its popularity in India. This is an inherent flaw of such trials, and limits the extrapolation of results to the entire subject population. However, this limitation does not reduce the external validity of the study because in practice only those subjects opt for a particular modality of treatment who have faith in its efficacy.

We chose, keeping the subjects' convenience and our infrastructure in mind, a 2-wk supervised out-patient intervention at IHC followed by 6 wk of closely monitored intervention at home. The 2-wk training period was adequate to make the subjects reasonably proficient in physical postures, breathing exercises, meditation and relaxation techniques, and to acquaint them with principles of stress management and a healthy yoga-based lifestyle. Leaving the subject to continue with these practices on his own and apply the principles learnt to everyday situations for 6 wk was reasonably long to test the feasibility of the intervention in real life, and to assess how far the gains achieved in 2 wk of supervised training were sustained during follow-up. Not only could the subjects continue with the intervention, the gains of the first 2-wk were improved upon during the 6-wk follow up.

The various interventions administered under the omnibus title 'yoga' cover a very wide spectrum. This is inevitable because yoga is not a system of medicine; it is a way of life, the implications of which go beyond health and disease[21]. When yoga is employed for prevention or management of disease, some convenient and highly visible elements of yoga are used selectively. Our intervention has used yoga as a tool in mind-body medicine[22]. This approach now has a strong scientific foundation in psychoneuroimmunology, and is particularly relevant to a disorder like bronchial asthma which is characterized by deranged immune function.

The results of the present study allow a few fairly firm conclusions. Significant, steady and progressive improvement in key objective variables such as PEFR and FEV_1 _only in the yoga group but not in the control group indicates the efficacy of yoga. This is further substantiated by the significantly greater improvement in quality of life in the yoga group than in the control group.

In yoga group, there was also a significant trend towards improvement in exercise tolerance assessed by the exercise-induced fall in FEV_1_, which became more evident in ES subjects. The effects of yoga on EIB in our study are similar to those reported by Jain *et al*. (1991)[5], who demonstrated a significant increase in exercise tolerance as indicated by a decrease in exercise-induced bronchial lability index (BLI) with yoga practice for 40-days in adolescents with mild to severe asthma and in another study by the same authors in moderate to severe chronic asthma patients[23].

Since the blood level of ECP is directly related to the severity of asthma and inversely related to lung functions, we have used ECP as a peripheral marker in understanding the disease progress [24,25]. In our present study, no significant changes were observed in ECP in either group. The high variability in ECP levels suggests the need for a much larger number of subjects for arriving at definitive conclusions.

Apart from the IgE mediated mast cell activation[26], mast cells may also be activated independently of high affinity IgE receptor (FcεRI), by emotional stress and it can induce asthma exacerbations [27,28]. The possibility is made more likely because psychological or emotional stress triggers dura mast cell secretion via release of corticotropin-releasing hormone, CRH[29]. Another study also indicated that acute psychological stress could lead to cardiac mast cell degranulation through CRH acting directly or through neurotensin[30]. Therefore, we speculated that yoga might play a vital role in reducing the mast cell activation levels by improving the emotional function of asthmatics thereby reducing the inflammatory condition and would cause the favourable effects on this outcome measure.

Another plausible explanation for the role of yoga in reducing the mast cell degranulation could be based on the frictional stress from air flowing through narrowed airways damaging the airway mucosa and thereby perpetuating airway inflammation and airway obstruction. At high air flow rates, high values of the frictional stress could damage the airway wall, especially during episodes of cough, and particularly when the mucosa is inflamed and friable as it is in asthmatic patients[31]. The slow and gentle breathing in some of the pranayamas (breathing practices in yoga) may reverse the process[32] by reducing the frictional stress, and thereby stabilizing the mast cell degranulation. Olness *et al*. (1999)[33] also demonstrated an inhibition of mast cell activation by conditioned relaxation techniques such as relaxation-imagery in children having migraine. In the present study, the urinary concentrations of 11β-PGF2α against exercise stimuli in yoga and control groups did not show any significant trend towards the inhibition of mast cell activation except a significant difference observed between groups at 4 wk in ES subjects (Table 5). Therefore, our study could not examine conclusively the above speculations because of high variability of 11β-PGF2α and a small sample size (yoga, 13; control, 9) in ES subjects. In addition, the exercise in the present study was a much less vigorous stimulus for provocation of airways and activating the mast cells than allergen provocation in which approximately 200–300% increase is seen in levels of urinary 11β-PGF2α with an allergen challenge[34].

The favourable effect of yoga in bronchial asthma observed by us in some outcome measures is consistent with similar results reported in several previous studies[3-5,10]. Most of these, except the study by Jain *et al *(1991)[5], were also randomized controlled trials. But lately there have been a few studies which have reported either negative or very weak effects of yoga[6-9,35]. All these were also controlled trials, and the study designs are apparently more rigorous than in the earlier trials. However, that does not necessarily mean that the positive results of earlier studies were due to insufficient rigor. Rather, it seems that in order to meet the exacting demands of methodology, some of the recent studies have overlooked some basic features of yoga. Moreover, yoga is much more than breathing exercises. It is only an integrated practice e.g. that given in the study by Nagarathna and Nagendra (1985)[10] or in the present study, which comes closest in an experimental situation to what yoga really is. In the study by Sabina *et al *(2005)[9], yoga has been equated with postures or breath work. The frequency is only twice a week without any serious monitoring of the frequency with which the practices are repeated at home on other days. Moreover, the control is a stretch. If yogic stretches are also treated like just a stretch, a non-yogic stretch could be equally good. It is only when the yogic stretch is performed with a yogic attitude, and preferably, when it becomes a component in the yogic way and view of life, that the yogic stretch acquires superiority over a non-yogic stretch[36]. That is possibly the reason why in the study by Sabina *et al *(2005)[9], both groups improved but the difference between the groups was not significant. In the most recent studies[8,9], the scientific rigor has been improved upon by giving to the control group an intervention similar to that given to the yoga group, and the trials have been masked. Masking in a study which involves highly visible physical practices is meaningless. Further, if the interventions in the two groups are very similar, the outcomes in the two groups cannot be very different. The mere fact that in one group the techniques have been selected from a yogic text and in the other from a text on physiotherapy or modern psychology will not affect the outcome. Further, the comprehensive approach to yoga has been avoided probably to keep the intervention well-defined. But in the process of testing a holistic treatment using a design which is ideal for testing a drug, the holistic treatment should not be reduced to one of its components. For the sake of fitting our design into a rigid framework, we should not end up evaluating a modality of treatment which is different from the one we have set out to examine. That answers also one of the commonest criticisms leveraged at studies like the present one i.e. the study does not allow us to determine the proportionate contribution of each component of the intervention. To view a holistic treatment as a combination of components is convenient but inappropriate. The efficacy observed is that of the whole intervention, and may be greater than the sum of the efficacy of its components.

The present study is the first one to attempt an investigation into the mechanism by which yoga helps bronchial asthma. Although it has not succeeded in throwing much light in that direction, there are several other subtle reasons why this study and many more similar studies in the future would be justified. Although there are several studies available on the efficacy of yoga in bronchial asthma and several other diseases, studies have to reach a critical mass before they can influence practice. Further, in case of yoga, very few of the studies are randomized controlled trials[1]. Because of the dilution of a few RCTs by several less rigorous trials, and also because of widespread scepticism with which disciplines like yoga are approached by practitioners of conventional medicine, the impact of the RCTs is inadequate. Further, it would help to integrate mind-body approaches like yoga into the practice of scientific medicine if the trials not only show that these approaches work, but also how they work in terms of measurable basic mechanisms.

## Conclusion

The present RCT has shown that adding a comprehensive yoga-based mind-body intervention to the conventional treatment improves several measures of pulmonary function in subjects having mild to moderate bronchial asthma. There is a significant trend suggesting a decrease in exercise-induced bronchoconstriction in the yoga group, particularly in the exercise-sensitive subjects. Yoga and control groups did not show a clear trend towards the inhibition of mast cell activation except for a significant difference observed between groups at 4 wk in exercise-sensitive subjects. Once again, in view of the high variability in response, a much larger number of exercise-sensitive subjects are required for definitive conclusions. Yoga improves the quality of life and reduces rescue medication use in bronchial asthma, and achieves the reduction earlier than conventional treatment alone. The trial supports the use of yoga in the management of bronchial asthma.

## Competing interests

The authors declare that they have no competing interests.

## Authors' contributions

RV conceived of the study, and participated in its design and coordination and carried out the entire study including data collection, statistical analysis and drafted the manuscript. RLB and KKD guided the study throughout right from its inception, participated actively in the design of the study data collection and statistical analysis, and conducted research committee meetings. They also helped draft the manuscript. All the authors have read and approved the final manuscript.

## Pre-publication history

The pre-publication history for this paper can be accessed here:


